# Phase-dependent iron depletion differentially regulates the niche of intestinal stem cells in experimental colitis via ERK/STAT3 signaling pathway

**DOI:** 10.3389/fimmu.2025.1537651

**Published:** 2025-01-30

**Authors:** Shubin Wang, Xiangjun Liu, Lu Xu, Jinyi Lang, Dengqun Liu

**Affiliations:** ^1^ Radiation Oncology Key Laboratory of Sichuan Province, Sichuan Cancer Hospital & Institute, Sichuan Provincial Engineering Research Center for Tumor Organoids and Clinical Transformation, Sichuan Clinical Research Center for Cancer, Sichuan Cancer Center, School of Medicine, University of Electronic Science and Technology of China, Chengdu, China; ^2^ Department of Experimental Research, Sichuan Cancer Hospital & Institute, Sichuan Provincial Engineering Research Center for Tumor Organoids and Clinical Transformation, Sichuan Clinical Research Center for Cancer, Sichuan Cancer Center, School of Medicine, University of Electronic Science and Technology of China, Chengdu, China

**Keywords:** deferoxamine, iron, intestinal stem cell, organoid, colitis

## Abstract

**Introduction:**

Ulcerative colitis (UC) is a global gastrointestinal disease, which is mainly caused by both dysfunctional epithelial barrier and inflammation response. Iron is a critical fundamental element for both the maintenance of homeostasis and the mediation of inflammation in many tissues. However, the role and mechanism of iron in the phase of enteritis and the subsequent repairing phase of intestinal stem cells has not been elucidated. In this study, we aimed to explore whether and how iron depletion would affect the occurrence and outcome of experimental colitis.

**Methods:**

Iron depletion was realized by deferoxamine (DFO) at either the early stage or late stage of dextran sulfate sodium (DSS) induced experimental colitis in mice. The gross images of colons, general health, histology, barrier integrity, and qRT-PCR were performed. Meanwhile, cell culture and colonic organoids were used to examine the influence of iron depletion *in vitro*. Signaling pathway and inflammatory infiltration were investigated by immunostaining.

**Results:**

Iron depletion within the early stage of DSS treatment significantly inhibited the onset of the inflammatory response, maintained the integrity of the colonic epithelium, and preserved the activity of intestinal stem cells (ISCs) both *in vivo* and *in vitro*. However, both continuous iron depletion by DFO and late DFO treatment aggravated colonic injury and postponed the recovery from colitis. Early DFO-induced iron depletion was able to maintain the p-STAT3 and p-ERK1/2 signaling pathways within the colonic epithelium at the early phase of colitis, but late DFO treatment inhibited the activity of these two pathways.

**Discussion:**

Our study demonstrated that the manipulation of iron depletion by DFO might greatly affect the outcomes of experimental colitis in a phase-dependent manner, which suggests that the balance of iron metabolism might be an effective therapeutic target for the clinical treatment of IBD patients.

## Graphical Abstract

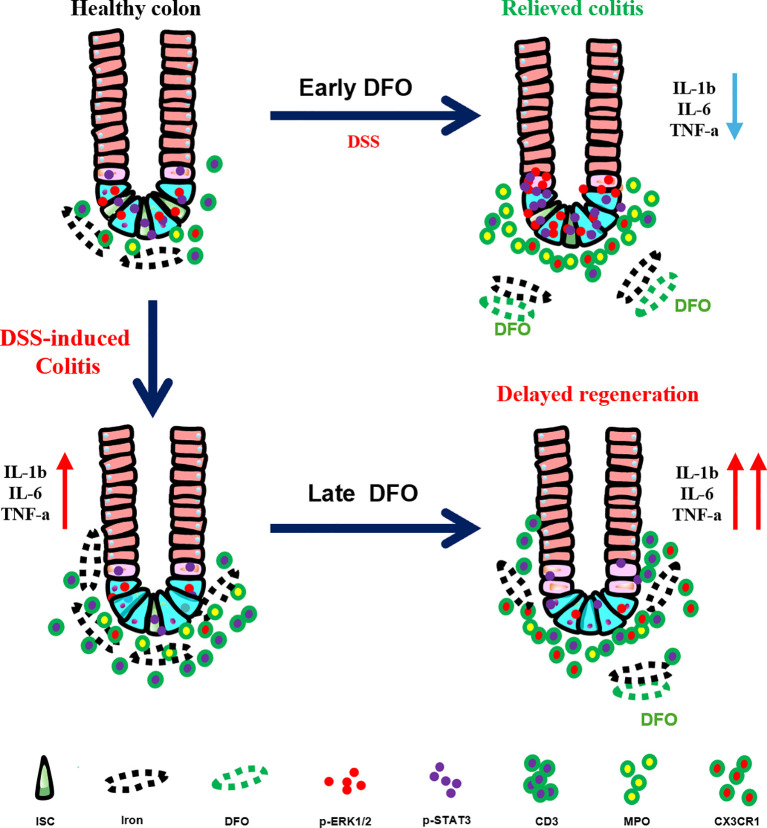

## 1 Introduction

Nutrition is a long-standing and vital topic in both physiology and pathophysiology. Among all nutrients, iron is a necessary micronutrient for many human tissues. Iron is absorbed by gastrointestinal epithelial cells, mainly in the small intestine, and it is stored mainly in the bone marrow, spleen, etc (1). Depending on the specific cellular status, iron can switch between its two forms, Fe^2+^ and Fe^3+^, which contributes to cellular adaptation in different physiological and pathological processes (2, 3). For example, there is a strong need for iron when energy production, proliferation, or deoxyribonucleic acid (DNA) synthesis are increased iron metabolism is indispensable for highly metabolic organs, such as the heart and skeletal muscles (4).

The balance of iron metabolism is crucial for both physiological health and a factor in the onset of many diseases. As mentioned above, cellular iron can switch between its two forms; therefore, iron overload usually results in high toxicity due to the formation of reactive oxygen species. For example, during the process of ageing, inflammation and iron status are strongly linked, which often causes anaemia accompanied by macrophage infiltration (5). Iron is also tightly linked to infection and immunity (6). When infection occurs, both invading pathogens and host cells, especially immune cells, demonstrate high iron requirements to sustain their functions, including metabolism and proliferation (7). Iron metabolism and inflammation in both the systemic and brain systems are also closely related to the progression of Parkinson’s disease (PD) (8). During inflammation, appropriate ferritin levels represent an important host defense mechanism that deprives bacteria of iron and protects tissue homeostasis. Both anaemia and hyperferritinaemia are harmful to human health (9). In acute lung inflammation, oxidative stress also results in iron accumulation in macrophages (10). Iron deposition is involved in the occurrence of pulmonary fibrosis in bleomycin (BLM)-treated mice and β-thalassemia-induced lung injury, and the administration of deferoxamine (DFO), which is an effective iron chelator, significantly reduces the degree of these two types of lung injury (11, 12). Therefore, targeting the balance of iron and inflammation in physiology and pathology could be a potential therapy for many clinical diseases (13).

Ulcerative colitis (UC) is a major inflammatory bowel disease (IBD) characterized by chronic relapsing inflammation and has severe negative impacts on the quality of life of patients. UC usually results in a great social burden and inconvenience to patients. Inflamed mucosal lesions in UC usually begin at the mucosal layer and gradually impair the colonic wall (14, 15). Currently, the development of UC is considered to be affected by complex interactions, including lifestyle, environmental factors, the gut microbiota, predisposing genetic background, and the immune system (16). Patients with IBD, even those in remission, must be screened regularly for malnutrition (17). The integrity of the colonic epithelium usually severely deteriorates due to inflammatory injury caused by an excessive immune response, and loss of integrity allows gut microbiota invasion, which exacerbates inflammation (18–20). Therefore, maintaining epithelial integrity is necessary for the treatment of UC and other intestinal diseases (21–24).

As mentioned above, iron participates in various biological processes, including oxygen transport, enzyme catalysis, photosynthesis and the formation of reactive oxygen species (ROS) (25, 26). Colitis is associated with disturbances in iron homeostasis that lead to a significant decrease in circulating iron concentrations (27, 28). Anaemia is observed in 25–60% of patients with UC and in bacteria mouse models, and decreased iron circulation and/or inflammation affect the recovery from colitis (29). Several previous reports have discussed the role of ferroptosis in colitis (30, 31), and a recent study showed deferasirox, which is an oral iron chelator tablet, alleviated DSS-induced colitis by improving gut microbiota (32). However, few studies have systematically investigated the exact role of iron manipulation by DFO in experimental colitis, especially in the different stages of colitis. Given the known effects of iron on inflammation and tissue recovery, we hypothesized that iron depletion by DFO might influence the occurrence and healing of dextran sodium sulfate (DSS)-induced colitis. In this study, we used two strategies for DFO administration to test this hypothesis, with the aim of revealing the specific impact of iron depletion by DFO at different stages of experimental colitis. We demonstrated that DFO-induced iron depletion resulted in different outcomes of colitis in a phase-dependent manner.

## 2 Materials and methods

### 2.1 Mice

Male C57BL/6 mice with 6- to 8-week-old age were purchased from GemPharmatech (Nanjing, China). B6;129S6-Gt(ROSA)26Sor^tm14(CAG-tdTomato)Hze^/J (tdTomato) and B6.129P2-Lgr5^tm1(cre/ERT2) Cle^/J (Lgr5-EGFP-IRES-CreERT2) mice were introduced from Jackson Laboratory (Bar Harbor, ME, USA), and Lgr5-EGFP-IRES-creERT2 mice were crossed with tdTomato mice to generate Lgr5-tdTomato progenies. The genotyping procedures were performed following the protocols provided by Jackson Laboratory. All the mice were housed in a specific pathogen-free (SPF) facility under a 12-hour light/dark cycle with free access to water and food, and the temperature and humidity in facility was monitored. All the animal treatments were complied with the guidelines for Care and Use of Laboratory Animals, and the experiment protocols were approved by the Ethics Committee of Sichuan Cancer Hospital and Institute (SCCHEC-04-2024-038).

### 2.2 DSS-induced experimental colitis and DFO treatment

Experimental colitis was induced as previously described. Briefly, healthy C57BL/6 mice or Lgr5-tdTomato transgenic mice were treated with free access to the drinking water, which was supplemented with 2.5% colitis grade dextran sodium sulfate (DSS) (#216011080, molecular weight: 36,000–50,000, MP Biomedicals) for 5 consecutive days or 7 consecutive days depending on different objectives. Deferoxamine mesylate (HY-B0988, MCE) was dissolved properly, and was given by daily intraperitoneal (i.p) injection at the different doses, including 25 mg/kg/day, 50 mg/kg/day, and 100 mg/kg/day. The administration of DFO for different purposes was specifically described in the related schematic illustrations. The number of mice in each study was described in the relevant figure legend. Three or more mice were included for each time point of different treatments.

### 2.3 Clinical symptom scoring of colitis

The severity of experimental colitis was evaluated by disease activity index (DAI). DAI is determined according to the combination of weight loss, stool consistency and rectal bleeding. For details, weight loss was scored ranging from 0-4, which was classified as none, 1–5%, 5–10%, 10–15%, and 15–20% weight loss. The consistency of stool was also scored ranging from 0-4, indicating normal stool, semi-normal stool, loose stool, loose stool adhered to anus, liquid stools adhered to the anus. Rectal bleeding was determined as follows: 0 = normal; 1 = seminormal; 2 = positive hemoccult; 3 = blood traces in stool visible; and 4 = gross rectal bleeding. These three parameters lead to a maximum of 12 scores for DAI.

### 2.4 Lineage tracing of Lgr5^+^ intestinal stem cells

In order to evaluate the protection of DFO on Lgr5^+^ ISCs and their progenies, Lgr5-tdTomato mice were used for the study (24). Briefly, mice were treated with DSS and DFO for 7 continuous days, and on day 6 mice were intraperitoneally injected with tamoxifen (2mg/20g) to activate Cre recombinase and label ISCs and progenies with tdTomato. Mice were sacrificed at day 7. Colon tissues were removed, flushed, and fixed for the preparation of frozen sections.

### 2.5 Tissue collection and H&E staining

Colonic tissues were collected at the indicated time as described respectively. Before sacrifice, mice were injected with 5′-bromo-2′-deoxyuridine (BrdU) (100 mg/kg) to label the proliferative cells for 90 min. Whole gastrointestinal tract was quickly collected and placed on ice. Gross images of colons were captured. Colons were flushed with ice-cold PBS and fixed with 4% precooled paraformaldehyde (PFA) (#BL539A, Biosharp) for 3 days, then further processed for dehydration and paraffin-embedding following the standard protocols in our laboratory. Sections of 4 μm thickness were dewaxed and rehydrated in gradient alcohols for subsequent hematoxylin-eosin (H&E) staining regularly.

### 2.6 Histopathological scoring

Swiss rolls of colonic tissues were used for histopathological analysis. Tissue images were independently observed by two individuals and scored as described previously (33). Briefly, distal colons were scored according to a scale that reflected different levels for the extent of inflammatory infiltration (0–5), crypt damage (0–4) and ulceration (0–3). Among them, inflammatory infiltration was scored as follows: 0 = no infiltration; 1 = occasional cell limited to submucosa; 2 = significant appearance of inflammatory cells in submucosa in focal sites; 3 = infiltration in both submucosa and lamina propria in focal sites; 4 = large amount of infiltrate in submucosa, lamina propria, and surrounding blood vessels; 5 = whole transmural inflammation. Crypt damage scores were determined as below: 0 = none; 1 = some crypt damage with spaces between crypts; 2 = larger spaces between crypts, loss of goblet cells, some shortening of crypts; 3 = large areas with loss of crypts but surrounding by normal crypts; and 4 = no remained crypts. Ulceration scores were given as follows: 0 = none; 1 = small, focal ulcers; 2 = frequent small ulcers; and 3 = large area lacking surface epithelium.

### 2.7 Goblet cell staining

Goblet cells were stained by Alcian blue and Periodic Acid-Schiff (PAS) staining. Briefly, tissue slides were processed as usual before staining. Alcian blue was stained for acid mucus polysaccharides with a Alcian blue kit (Catalog: #E670107, BBI). PAS staining was used to stain neutral mucus substances by a PAS Stain Kit with Hematoxylin (Catalog: G1281, Solarbio). All the staining procedures were performed following the protocols provided by the manufacturers. Nuclei were stained with fast red or hematoxylin solution.

### 2.8 Immunological staining

Colonic tissue slides were processed using xylene and serial ethanol routinely. After rehydration, antigen retrieval was performed for 20 min in boiling TRIS-EDTA Antigen Retrieval Solution (#BL618A, Biosharp), then antigen blocking was conducted with 1% bovine serum albumin (BSA, #A7906, Sigma-Aldrich) solution, containing 0.5% Triton X-100. Primary antibodies, including rabbit, rabbit anti-Ki67 (Abcam, UK), mouse anti-BrdU (Biolegend, USA), rabbit anti-ZO-1 and rabbit anti-Occludin (Proteintech, China) were diluted at 1:200, and then incubated overnight at 4°C. On the second day, primary antibodies were removed, and after washing slides, HRP-linked secondary antibody (ZSBio, Beijing, China) was incubate for 40 min. DAB kit (ZSBio, Beijing, China) was used for immunohistochemical (IHC) visualization. Hematoxylin was used for counterstaining to show nucleus. For immunofluorescent (IF) staining, AlexFluo™ 594 labelled highly cross-adsorbed donkey anti-rabbit or mouse IgG (H+L) antibodies (ThermoFisher, USA) were used, and nuclei were stained with 4’,6-diamidino-2-phenylindole (DAPI) (Vector, Burlingame, CA).

### 2.9 Cell culture and treatment

Caco-2 and HCT-116 were widely used cell models to mimic human colonic epithelial cells (34, 35). These two cell lines were obtained from Cell Bank of the Chinese Academy of Sciences (Shanghai, China) and kept in our laboratory. Briefly, cells were cultured in the required basal medium, which was supplemented with 10% FBS and 1% penicillin/streptomycin solution. Cell culture was performed in the incubator (Thermofisher, USA) with 5% CO_2_ at 37°C. Cells were cultured to form confluent monolayer epithelium, and then were treated with lipopolysaccharide (LPS) (100ng/ml) or 1mM hydrogen peroxide to induce inflammation and oxidative stress. DFO was loaded simultaneously with the stimulation at the concentrations of 50 μM or 100 μM. All the drugs were incubated with Caco-2 or HCT-116 for 24 h. Cell viability was determined by CCK-8 kit (Dojindo, China). For staining, cells were fixed with 4% PFA for 30 min, washed with cold PBS, and stained for ZO-1 and Occludin.

### 2.10 ROS detection

The intracellular level of reactive oxygen species (ROS) was detected using a dihydroethidium (DHE) probe (S0063, Beyotime, China) following the manufacture’s protocol. Briefly, DHE was dissolved in DMSO, and loaded into the culture medium at a final concentration of 5 μM. Cells were incubated with DHE probe at 37°C for 30 min before observation.

### 2.11 Isolation of colonic crypts and organoid culture

Colonic tissues were freshly collected from healthy, DSS-treated and DFO-treated mice as described in each experiment. After flushing with ice-cold PBS, colons were opened longitudinally on ice, cut into 3 to 5 mm pieces, washed vigorously washed in PBS to remove bacteria, and finally placed into chelation buffer, which contained 5 mM EDTA (25,300,096, Invitrogen) and 1% FBS. Tissues were incubated for 30 min at 4°C, and then washed with cold PBS to remove EDTA. Crypts were isolated by vigorous shaking. Individual colonic crypts in the supernatant were collected by centrifugation at 4°C and 800×g for 3 min. Crypts mixed with Matrigel (#354230, Corning, USA) were seeded into 96-well fat-bottom plates to culture enteroids using IntestiCult™ Organoid Growth Medium (#06005, STEMCELL Technologies, Canada) supplemented with 100 μg/mL streptomycin and 100 units/mL penicillin.

### 2.12 Image acquisition and data collection

Images of H&E and IHC staining of slides and of organoids in culture plates were captured by M5000 (TermoFisher, USA), Cytation 5 (BioTek, USA) or BX53 (Olympus, Japan) microscope. Image parameters were analyzed by ImageJ (NIH, USA) for each image. IF staining images were captured by a A1R confocal microscope (Nikon, Japan) or Zeiss Axio Observer with Apotome3. Each value was calculated based on at least three independent replicates.

### 2.13 RNA isolation and qRT-PCR assay

Total RNA was isolated from mouse colon tissues or cultured cells using RNAiso (9109, TaKaRa, Japan) following the manufacturer’s recommendations. The quality and concentration of RNA samples were determined using a NanoDrop2000 spectrophotometer (Thermo Scientific). Reverse transcription was performed using Hifair II 1st Strand cDNA Synthesis SuperMix (11137ES60, YEASEN, China). qPCR was conducted using Hieff qPCR SYBR Green Master Mix (11203ES08, YEASEN) using a C1000 instrument (Bio-Rad, USA). Primer sequences used in this study were listed in **Supplementary Table S1**. Gene expression results were normalized to that of β-actin, and relative expression was determined by the 2−ΔΔCt method.

### 2.14 Statistical analysis

All the data were presented as mean ± SD, and analyzed with GraphPad Prism 9 (GraphPad Software, USA). Comparison between two different groups was conducted by two-tailed unpaired Student’s t-test or non-parametric test depending on whether the data conformed to normal distribution. Comparing of multiple groups was analyzed using one-way ANOVA with *post-hoc* Tukey’s test. P values less than 0.05 were considered as statistically significant different (*: P <0.05, **: P <0.01, ***: P <0.001, ****: P <0.0001). P >0.05 was considered as nonsignificant (n.s).

## 3 Results

### 3.1 DFO caused iron depletion postponed the onset of experimental colitis

**Figure 1:**
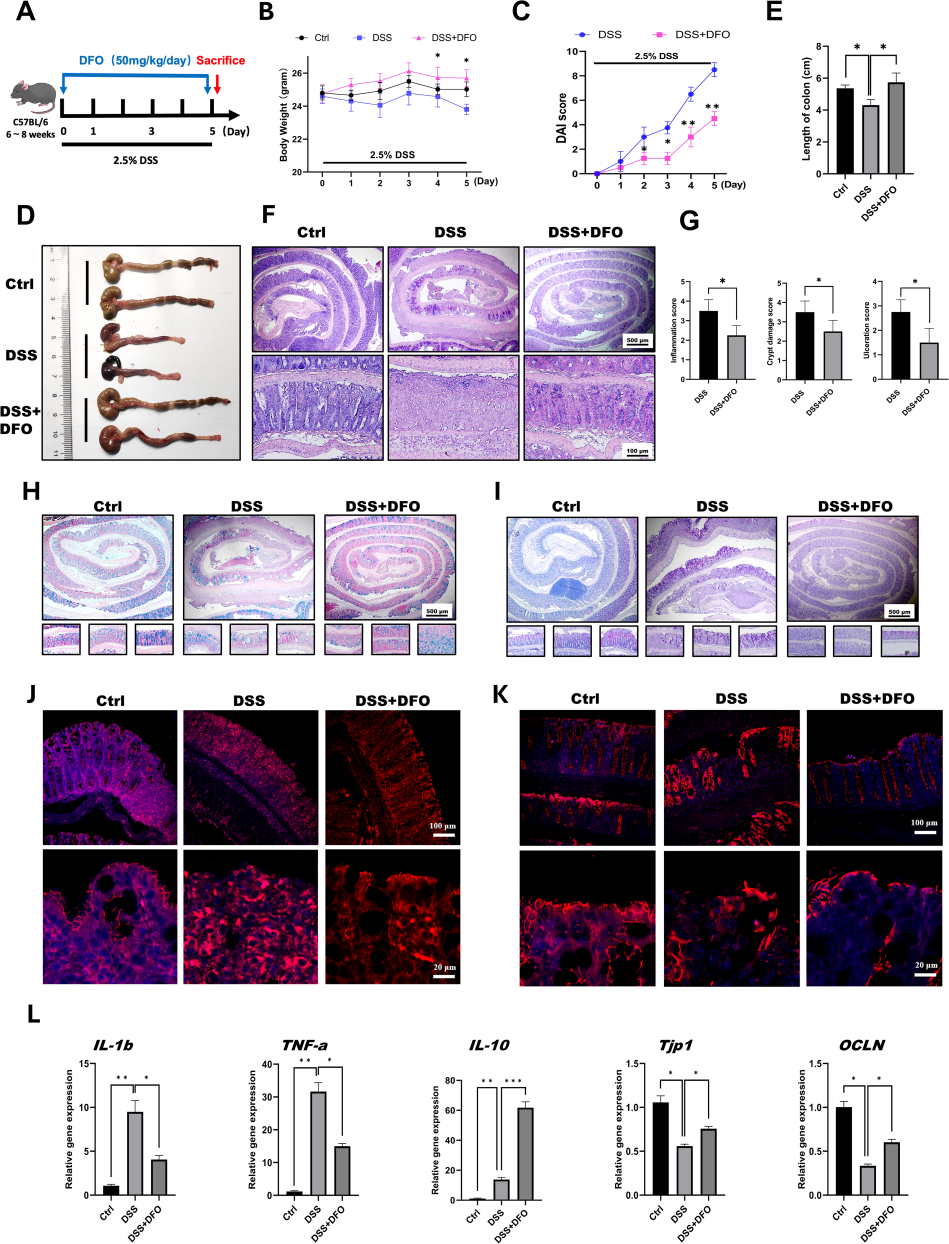
Iron depletion by DFO significantly mitigated early colonic mucosal injury in mice with DSS-induced colitis. **(A)** Schematic diagram of the establishment of DSS-induced colitis and 50 mg/kg/day DFO treatment. **(B)** Weight loss of mice in the DSS group and the DSS+DFO group (n=4 per group). **(C)** Comparison of disease activity index (DAI) scores between DSS-treated mice and DSS+DFO-treated mice (n=4 per group). **(D)** Representative gross images of the colons of mice in the healthy control, DSS, and DSS+DFO groups. **(E)** Statistical analysis of the length of colons in different groups. **(F)** Representative H&E images of colons from healthy controls, the DSS group and the DSS+DFO group. **(G)** Histological scores indicating that inflammation, crypt damage, and ulceration were lower in the DFO group than in the DSS group. **(H)** Alcian blue staining images. **(I)** PAS staining of colonic tissues from different groups (including Swiss roll, distal, middle, and proximal colonic images). **(J)** IF images of ZO-1 staining in different groups. **(K)** IF images of Occludin in the two groups. **(L)** Relative gene expression levels of IL-1b, TNF-a, IL-10, TJP-1, and OCLN were examined via qRT-PCR. *: P < 0.05, **: P < 0.01, ***: P < 0.001.

According to our hypothesis, it is important to determine the protective effects of iron depletion by DFO. So, at the beginning of this study, we firstly evaluated the potential influence of iron depletion on the pathogenesis of 2.5% DSS-induced colitis. Since 50mg/kg DFO was able to alleviate pulmonary fibrosis (12), we referred to such dose at the beginning of this study. DFO was given by daily intraperitoneal injection at the dose of 50 mg/kg/day during the administration of DSS for 5 consecutive days (**Figure 1A**). Interestingly, it was found that mice in DFO-treated group had significantly less weight loss than those mice only received DSS (**Figure 1B**). Disease activity index (DAI) is widely used to describe the severity of colitis, and we found that DFO treatment significantly decreased DAI scores causing by DSS treatment (**Figure 1C**). When we collected the colon samples, it was observed that the gross appearance of colons in DFO-treated mice was much better than that of DSS group, DFO-treated colons were similar to healthy control ones (**Figure 1D**). Statistical analysis showed that the mean length of colons in DFO group was statistically longer than that of DSS-treated mice (**Figure 1E**). H&E images demonstrated that DSS caused serious damage of colonic epithelium, and DFO obviously alleviated DSS-induced early colitis (**Figure 1F**). Histological scores, including inflammation score, crypt damage score, and ulceration score, showed that there was a significant alleviation of mucosal damage in DFO group compared to DSS group (**Figure 1G**). And there were more goblet cells in colonic epithelium of DFO treated mice as shown by Alcian blue (**Figure 1H**) and PAS staining (**Figure 1I**). Immunofluorescent images demonstrated that there were more ZO-1 (**Figure 1J**) and Occludin (**Figure 1K**) signals in DFO-treated colonic epithelium as compared with DSS group, indicating better integrity of intestinal barrier (**Figure 1J, K**). In addition, gene expression levels of IL1b, TNF-a, IL-10, TJP-1, and OCLN were consistent to the previous histological changes (**Figure 1L**). Therefore, these data suggested that DFO treatment during the early stage could significantly alleviated DSS-induced colitis.

### 3.2 Different doses of DFO exhibited similar protection on early mucosal injuries

**Figure 2:**
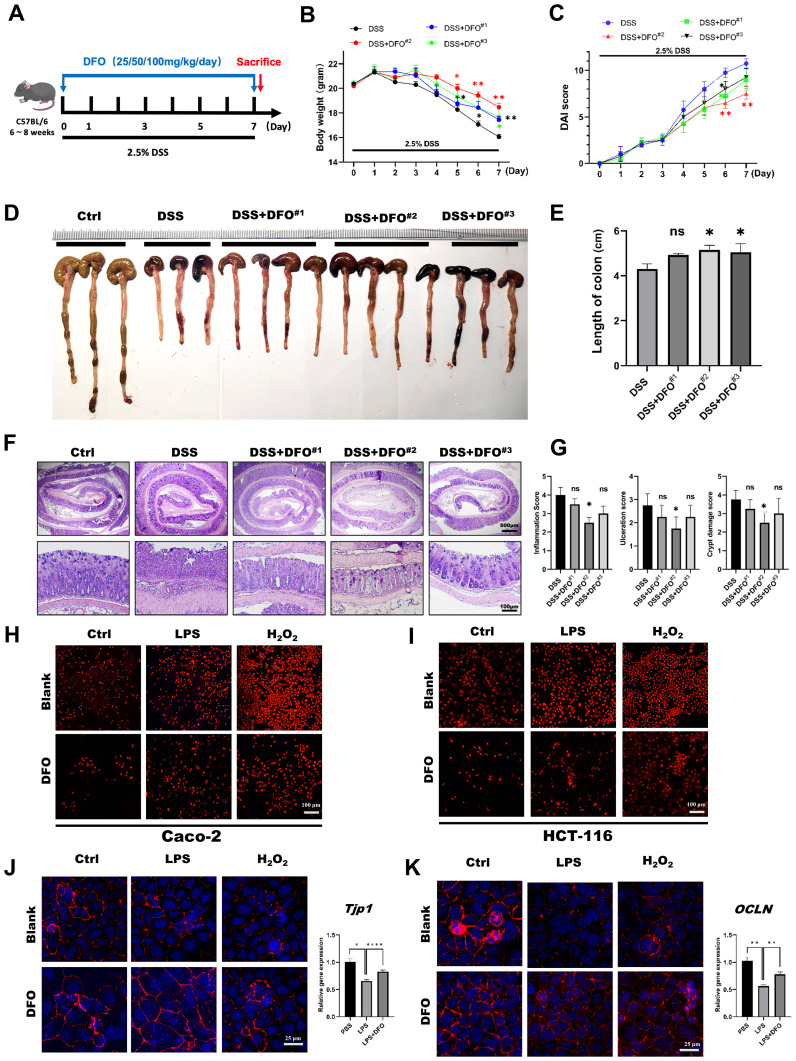
The protective effects of iron depletion by DFO exhibited a dose-effect response. **(A)** Experimental schematic diagram of treatment with different concentrations of DFO during DSS-induced colitis. **(B)** Weight loss of mice in different groups (DFO#1: 25 mg/kg/day; DFO#2: 50 mg/kg/day; DFO#3: 100 mg/kg/day) (n=4 per group). **(C)** Comparison of the disease activity index (DAI) scores of the mice in the different groups. **(D)** Representative gross image of colons from mice in each group. **(E)** Quantification of the length of colons in different groups. **(F)** Representative H&E images of colons. **(G)** Histological evaluation of inflammation, crypt damage, and ulceration in each group. **(H, I)** DHE staining revealed that DFO inhibited the level of ROS in Caco-2 **(H)** and HCT-116 **(I)** cells after LPS or H_2_O_2_ treatment. **(J)** DFO increased the intensity of ZO-1 staining and the gene expression level of TJP-1 in Caco-2 cells after LPS and H_2_O_2_ treatment. **(K)** DFO treatment increased the fluorescence intensity of Occludin and the mRNA level of OCLN in Caco-2 cells. *: P < 0.05, **: P < 0.01, ****: P < 0.0001.

Due to the previous basic discovery, we want to further confirm the protective effects of DFO-induced iron chelation on colonic epithelial injuries. Since DFO was an effective chelator of iron, and its clearing patch was tightly related to the doses. Therefore, we speculated that whether different doses of DFO had different protective effects on DSS-induced colitis. In order to validate this hypothesis, we treated mice with three different doses of DFO (25 mg/kg/day, 50 mg/kg/day, and 100 mg/kg/day respectively) for 7 consecutive days (**Figure 2A**). Interestingly, we found that mice in all the three groups had an alleviation in the terms of less weight loss, lower DAI scores and longer length of colons. And the most effective prevention was observed in mice which received a dose of DFO at 50mg/kg/day (**Figures 2B–E**). Histological analysis revealed that the most ideal dose of DFO was 50mg/kg/day (**Figures 2F, G**). We also validated the protection of DFO using Caco-2 and HCT-116 cell lines. Similarly, it was observed that DFO decreased the contents of ROS as shown by DHE staining (**Figures 2H, I**). In addition, DFO alleviated the loss of ZO-1 and Occludin caused by LPS and H_2_O_2_ in Caco-2 cell line (**Figures 2J, K**), and different doses of DFO exhibited similar protective effects in DSS-treated colonic tissues (**Supplementary Figures S1A, B**). DFO also rescued the mRNA levels of Tjp1, OCLN, and Lgr5 in LPS treated Caco-2 cell line (**Supplementary Figure S1C**), indicating better integrity of intestinal barrier. And here were more goblet cells in colonic epithelium of DFO treated mice as shown by Alcian blue (**Supplementary Figure S1D**) and PAS staining (**Supplementary Figure S1E**). These results indicated that iron depletion in an appropriate extent was capable to alleviate early mucosal injury in experimental colitis.

### 3.3 DFO-induced iron depletion preserved the activity of ISCs in the circumstance of experimental colitis

**Figure 3:**
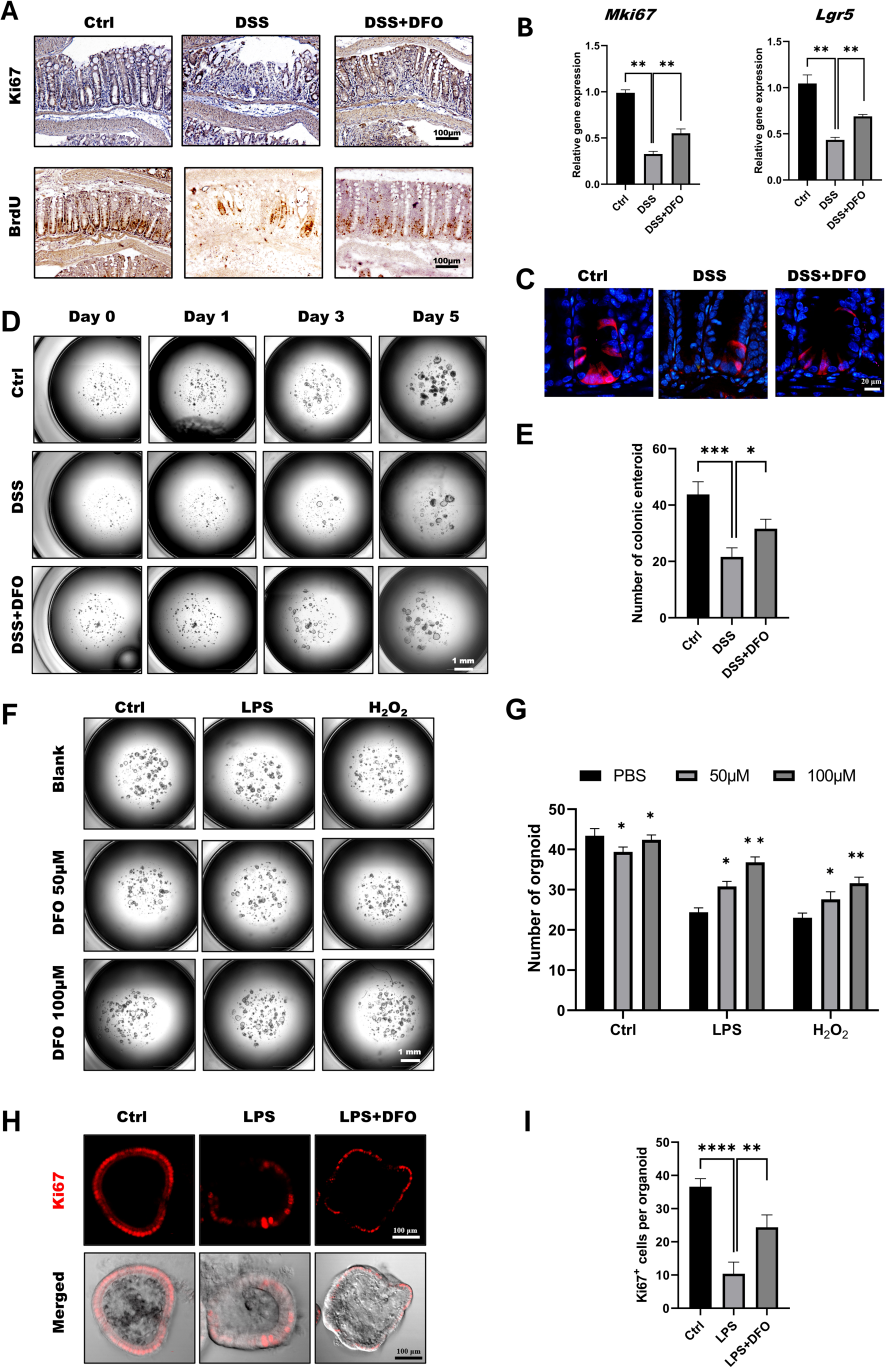
Iron depletion by DFO preserved the viability of colonic ISCs. **(A)** Representative IHC images of Ki67 and BrdU staining in different groups. **(B)** qPCR results showing that DFO increased the gene expression levels of Mki67 and Lgr5 in colonic tissues after DSS-induced injury. **(C)** Lineage tracing of tdTomato^+^ progenies derived from Lgr5^+^ ISCs in different groups. **(D)** Representative images of enteroids cultured from the colonic crypts of different groups. **(E)** Statistical analysis of the number of colonic enteroids counted on day 5 after culture. **(F)** Representative images of colonic enteroids treated with 50 μM and 100 μM DFO after the administration of LPS or H_2_O_2_. **(G)** Quantification of the number of organoids in different groups after DFO treatment. **(H)** *In situ* IF staining for Ki67^+^ proliferative cells within enteroids after LPS and DFO treatment. **(I)** Statistical analysis of Ki67^+^ cells per organoid. *: P < 0.05, **: P < 0.01, ***: P < 0.001, ****: P < 0.0001.

According to the previous results, we tried to explore the mechanisms of how DFO-induced iron depletion would exhibit the function of epithelial protection. Since ISCs are the basis for the generation of newly formed colonic epithelia cells, we wondered how iron depletion by DFO would affect the activity of colonic ISCs. Using IHC staining against BrdU and Ki67, we found that there were more Ki67 and BrdU positive cryptal epithelial cells in DFO-treated mice at day 5 in DSS-induced colitis (**Figure 3A**). Meanwhile, qRT-PCR data demonstrated that the relative mRNA expression levels of MKi67 and Lgr5 were much higher in DFO group as compared to mice which only received DSS treatment (**Figure 3B**). We also examined the differentiation of Lgr5^+^ ISCs by lineage tracing using Lgr5-tdTomato mice. Similarly, it was noticed that mice in DFO group had more tdTomato^+^ progenies within colonic crypts than those in DSS group (**Figure 3C**). Enteroid culture is widely used to reflect the activity of ISCs (36), and we also isolated colonic crypts of mice in different groups. It was observed that DSS-treated crypts formed less and smaller colonic enteroids than healthy crypts, but the enteroids from DFO-treated mice grew much better compared to DSS group (**Figures 3D, E**), suggesting that a higher activity of colonic ISCs was preserved due to the administration of DFO. In addition, we also cultured colonic enteroids using healthy mice, modeled the inflammatory injury using LPS and H_2_O_2_, and tested the protection of DFO from such an injury. Interestingly, we found that DFO at the concentration of 50 μM and 100 μM could alleviate LPS and H_2_O_2_ caused damage to colonic enteroids (**Figures 3F, G**). Meanwhile, there were more Ki67 positive proliferative epithelial cells in DFO treated enteroids than those enteroids received LPS only (**Figures 3H, I**). So, these results showed that during the early stage of experimental colitis, iron depletion by DFO could preserve the activity of colonic ISCs at the front of inflammatory stimulation.

### 3.4 Consecutive iron depletion impaired the recovery of experimental colitis

**Figure 4:**
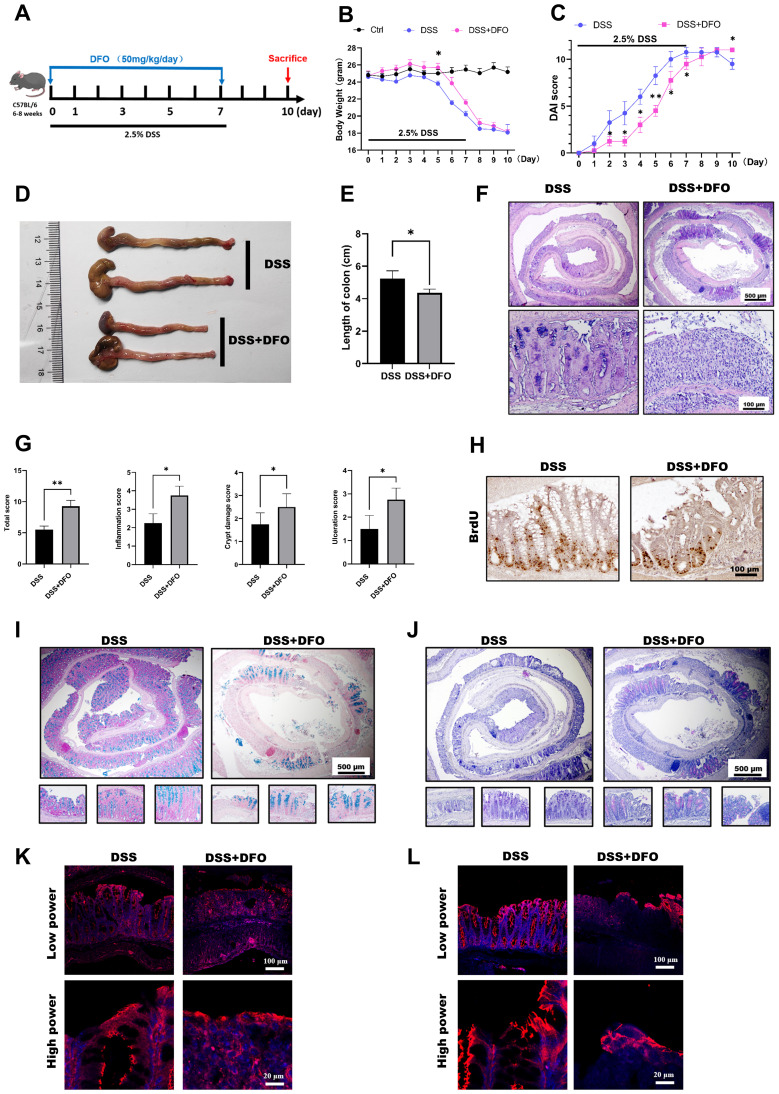
Continuous iron depletion inhibited the recovery of the colonic epithelium from colitis-induced injury. **(A)** Schematic diagram of continuous iron depletion by DFO. **(B)** Loss of weight of the mice in different groups (n=4 per group). **(C)** Comparison of disease activity index (DAI) scores between DSS-treated mice and continuous DFO-treated mice. **(D)** Representative gross images of colons from mice in different groups. **(E)** Comparison of colon length among the different groups. **(F)** Representative H&E images of colonic tissues from the DSS group and continuous DFO group. **(G)** Comparison of histological scores between the DSS group and continuous DFO-treated group. **(H)** Representative BrdU IHC images of the two groups. **(I)** Alcian blue staining of colons from the two groups. **(J)** PAS staining of goblet cells in different groups. **(K)** IF images of ZO-1 staining. **(L)** Representative IF images of Occludin in these two groups. *: P < 0.05, **: P < 0.01.

Those above results showed an affirmative protection of iron chelation by DFO on DSS-induced colitis, but colitis is a continuous process including injury and regeneration of colonic epithelium. Since different doses of DFO similarly protected the early mucosal injury in DSS-induced colitis, we further examined how iron depletion by DFO would affect the recovery of experimental colitis. Mice were treated by 2.5% DSS in the drinking water for 7 days with or without DFO, and they were monitored for another 3 days of recovery (**Figure 4A**). Surprisingly, we found that continuous iron depletion by DFO caused a significant loss of body weight at day 10, which was similar to that of DSS group (**Figure 4B**). The DAI scores in DFO group at day 10 were even worse than the scores in DSS group (**Figure 4C**). The gross images and lengths of colons showed that mice after consecutive iron depletion had a worse recovery (**Figures 4D, E**). Histological analysis showed that there were less regenerated colonic crypts and less BrdU positive proliferating epithelial cells in DFO group (**Figures 4F–H**). Accordingly, Alcian blue and PAS staining showed there were less mucus-secreting goblet cells (**Figures 4I, J**), and there were also fewer fluorescent signals of tight junction proteins, including ZO-1(**Figure 4K**) and Occludin (**Figure 4L**) in DFO group. Therefore, these results indicated that consecutive iron depletion by DFO inhibited the recovery of colonic epithelium from DSS-induced colitis.

### 3.5 Different strategies of iron deprivation by DFO exhibited different outcomes for the regeneration of DSS-induced colitis

**Figure 5:**
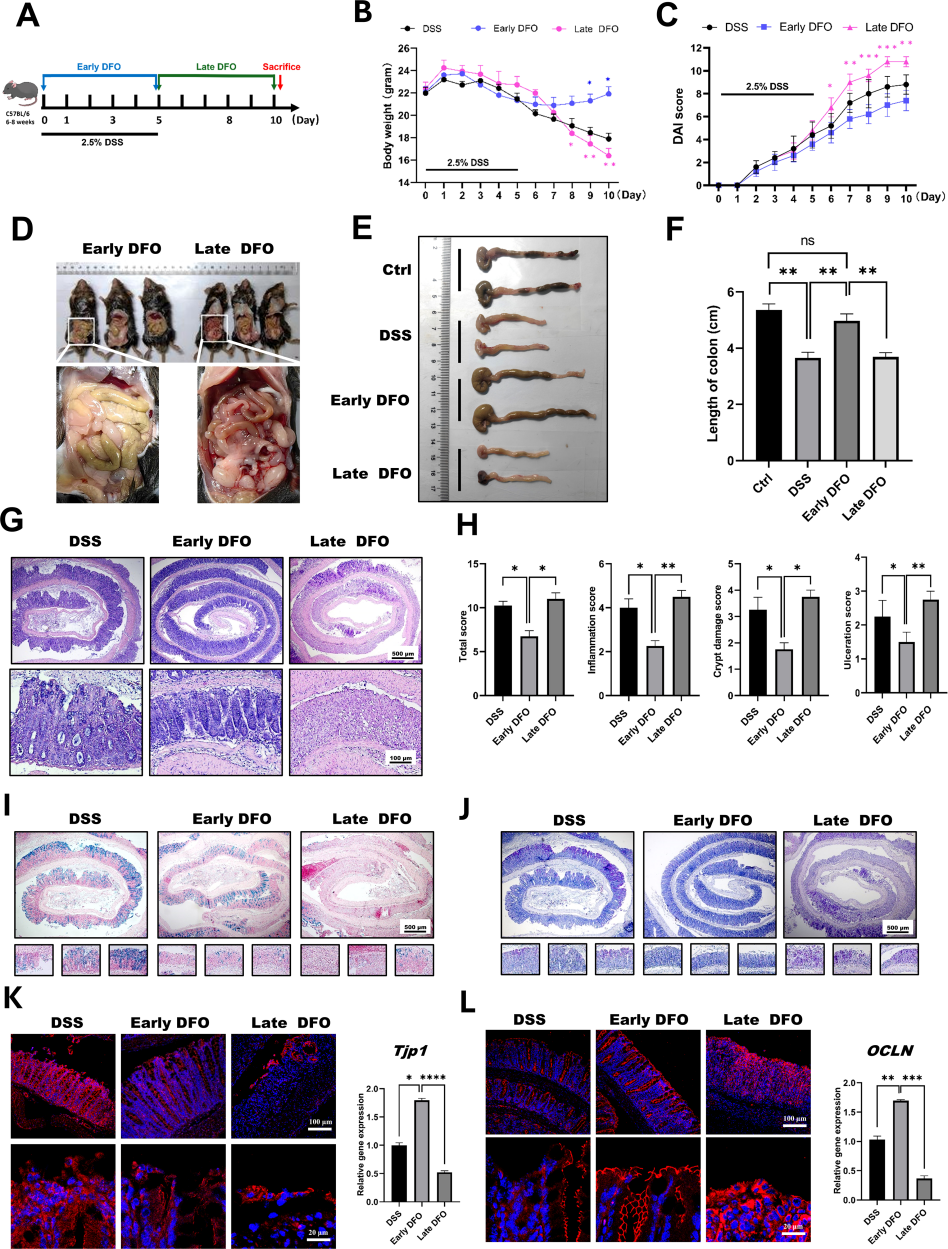
Iron depletion by DFO resulted in differential outcomes depending on the phase of colitis. **(A)** Schematic diagram for the different strategies of iron DFO by early DFO and late DFO. **(B)** Weight loss among the different groups (n=5 per group). **(C)** Comparison of disease activity index (DAI) scores between different DFO treatment groups and the DSS control group. **(D)** Comparison of gross images of the gastrointestinal tract *in vivo* on day 10 after DSS-induced colitis. **(E)** Representative images of isolated colons from different groups on day 10. **(F)** Statistical analysis of the length of the colons in each group (n=5). **(G)** Representative H&E images of colonic tissues from each group. **(H)** Histological scores of inflammations, crypt damage, and ulceration in each group (n=5). **(I)** Alcian blue staining images. **(J)** PAS staining of colonic tissues from different groups. **(K)** IF images of ZO-1 staining and the results of qPCR analysis of TJP-1 expression. **(L)** IF images of Occludin and qPCR data of OCLN expression levels in the different groups. *: P < 0.05, **: P < 0.01, ***: P < 0.001.

The above results demonstrated that iron depletion by DFO alleviated the onset and inhibited the recovery of experimental colitis respectively, so we speculated that whether the different strategies of iron depletion might demonstrate different outcomes for the epithelial regeneration in DSS-induced colitis. Therefore, we conducted iron depletion by DFO at the early stage and late stage respectively, which was shown in **Figure 5A**. As expected, it was identified that both loss of body weight and DAI scores showed that mice received early DFO treatment exhibited a better recovery at day 10 than those received the late DFO treatment (**Figures 5B, C**). The gross images also revealed that by the end of this study, the early DFO treatment reduced hemorrhage (**Figure 5D**), increased the length of colons compared with DSS with mice in DSS group and late DFO group (**Figures 5E, F**). We further examined the histological differences by H&E, Alcian blue and PAS staining. It was found that the histological characteristics of colon tissues in early DFO group had a better outcome, including more regenerated colonic crypts, less inflammation and ulceration, more Alcian blue positive and PAS positive goblet cells, and qPCR also revealed a decreased level of inflammation (**Figures 5G–J**). Notably, the epithelial tight junction in early DFO group showed more fluorescent signals and higher mRNA levels of ZO-1 and Occludin (**Figures 5K, L**), indicating that early DFO administration increased the barrier integrity of colonic epithelium. Therefore, these results supported that different patterns of iron depletion by DFO could significantly affect the regenerating outcomes of colonic epithelium after DSS induced inflammatory injury.

### 3.6 DFO-induced iron depletion changed signaling transduction within epithelial cells and dynamically affected infiltration of immune cells

**Figure 6:**
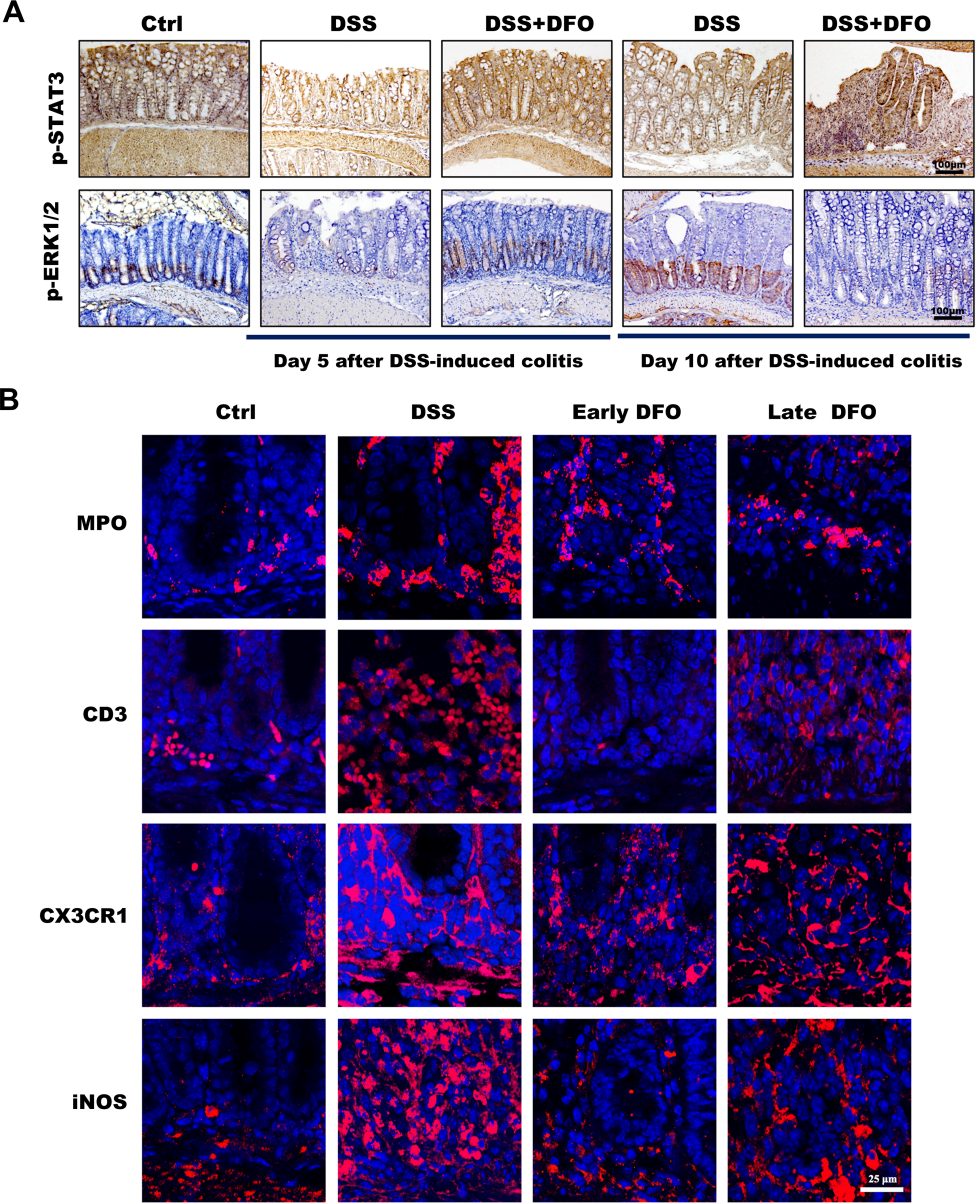
Different iron depletion strategies influenced signaling transduction within the colonic epithelium and the infiltration of inflammation-related immune cells. **(A)** Representative images of IHC staining with anti-p-STAT3 and anti-p-ERK1/2 antibodies in different colonic tissues (Bar = 100 μm). **(B)** Immunofluorescence (IF) staining of different DFO-treated colonic samples to show the infiltration of different subtypes of immune cells. MPO: neutrophils, CD3: Tcells, CX3CR1 and iNOS: macrophages (Bar = 25 μm).

As described in the above results, iron depletion could differentially affect the onset and recovery of DSS-induced colitis. It is useful to clarify the potential mechanisms for developing prospective therapeutic methods against UC. To clarify why DFO-induced iron depletion differentially affected the process of experimental colitis, we further examined the possible molecular pathways and infiltration of immune cells at the onset and recovery stages of colitis. STAT3 and ERK1/2 signaling pathways are critical molecules which can modulate the signaling transduction and proliferation activity of ISCs (24). Therefore, we performed staining of p-STAT3 and p-ERK1/2 to evaluate the changes in the activity of STAT3 and ERK1/2 pathways, respectively. It was found that colonic epithelium had a significant decrease of both p-STAT3 and p-ERK1/2 at day 5 after DSS treatment, the administration of DFO during the early stage of colitis reserved the expression of p-STAT3 and p-ERK1/2. And at day 10 of colitis the colonic epithelium showed an apparent recovery of p-STAT3 and p-ERK1/2 in DSS group compared to day 5. However, the late DFO treatment significantly inhibited this recovery (**Figure 6A**). Meanwhile, we also stained MPO to label neutrophils, CD3 to label T cells, CX3CR1 and iNOS to label macrophages using colonic tissues. Surprisingly, it was demonstrated that there were significantly fewer neutrophils, T cells and macrophages in the lamina propria of colons in the early DFO treated mice compared to DSS group, but the late DFO treatment caused similar infiltration of these immune cells to DSS group (**Figure 6B**). These data suggested that iron depletion by DFO at the early stage of colitis could preserve the activity of ISCs and inhibit the inflammation, but the late iron depletion inhibited the recovery of experimental colitis.

## 4 Discussion

Due to the increasing morbidity with social development, UC is attracting more scientific attentions during the past decades. UC brings the patients many inconveniences, and it costs more and more healthcare resources (37). Therefore, it is of great value to reveal the exact pathological mechanisms and develop the effective countermeasures of UC (38, 39). Iron is an important element for the maintenance of tissue and cellular homeostasis, and the impacts of iron balance is critical to the intestinal mucosal homeostasis and inflammation (40). Unfortunately, the metabolic balance of iron has not been well investigated during the process of UC. Deferoxamine (DFO) is widely used in the field to chelate intracellular iron for the purpose of investigating the role of iron in cellular processes (41). It binds both free plasma iron and excess iron within cells (42). In the current study, we demonstrated that iron depletion by DFO could affect the clinical and pathological characteristics of experimental colitis, whose administration and outcomes was tightly related to the phase of disease.

The onset of DSS-induced experimental colitis happens after 5~7 days of DSS treatment. Both of previous reports and our observation have found that DSS induced colitis started from the distal colon (43, 44). At this stage, the integrity of colonic epithelium was not greatly disrupted. However, the immune cells including macrophages, T cells, and neutrophils were stimulated by DSS, and they were recruited into the lamina propria of colons. Iron would contribute to the generation of excessive reactive oxygens species (ROS). It was found that iron depletion by DFO greatly inhibited the onset of colonic inflammation, which was confirmed by less loss of body weight, DAI score, better gross and histological appearance (**Figures 1B–K**). And qRT-PCR data also supported that the administration of DFO significantly alleviated the inflammatory responses (**Figure 1L**). In addition, we evaluated the dose response of iron depletion on the inhibition of early colitis using three different doses of DFO (25,50, 100 mg/kg/day respectively). The data showed that the protective effects of DFO on DSS-induced colitis had an apparent dose effect response (**Figures 2B–G**).

While the administration of DFO inhibited the onset of colonic inflammation, iron depletion also greatly preserved the activity of ISCs. Both IHC staining against BrdU, Ki67 and qRT-PCR showed DFO treatment saved more ISCs. Importantly, loss of Lgr5^+^ ISCs happened at the early stage of colitis. We observed more tdTomato^+^ labelled cells derived from Lgr5^+^ ISCs in DFO treated transgenic mice (**Figure 3C**). Furthermore, using ex vivo culture of colonic enteroids, we found colonic crypts derived from DFO treated mice generated more colonic organoids compared to DSS treated mice (**Figures 3D, E**). Since DFO inhibited the inflammation *in vivo*, we also examined whether it could show similar protection *in vitro*. We cultured colonic cell lines, including Caco-2 and HCT-116. Although these two cell lines are derived from human colorectal cancers, they have a typical colonic epithelial cell-like morphology, and thus they are frequently used as an *in vitro* model to study epithelial integrity (45–48). After the stimulation of LPS and H_2_O_2_, the administration of DFO was capable to block the increase of ROS and maintain the expression of ZO-1 and Occludin (**Figures 2H–J**, **Supplementary Figures S1A–C**). In addition, it was demonstrated that DFO treated organoids suffered from an alleviated injury caused by LPS and H_2_O_2_, and they had more Ki67^+^ proliferative epithelial cells (**Figures 3H, I**). Therefore, iron depletion could postpone the onset of DSS-induced inflammation, and the administration of DFO could also directly mitigate the inflammatory injury to colonic epithelium and ISCs.

The main problem is the inflammation response and colonic epithelial injury at the early stage of experimental colitis (44). However, during the late stage, the major aspect has been changed to the regeneration of damaged epithelium (49). In the continuous iron depletion model by DFO, it was observed that DFO treated mice had worse recovery in contrast to those control mice. For instance, they had shorter length of colons, fewer BrdU^+^ cryptal cells, less Alcian blue or PAS positive goblet cells, and decreased epithelial integrity stained by ZO-1 and Occludin. To further validate these results, we adopted two iron depletion strategies to treated mice with experimental colitis, including early DFO and late DFO. The data supported our hypothesis that early DFO greatly alleviated the injury to colonic epithelium thus mice had better outcomes at day 10. However, mice received late DFO treatment had worse recovery compared to DSS only group. Review of European studies of adults with Crohn’s disease (CD) or UC reported that 57% of the patients with anemia were iron deficient, while an earlier analysis found that iron deficiency was observed in 36–90% of all patients with CD (50, 51). Iron deficiency might be a possible reason for the insufficient epithelial recovery. Therefore, we concluded that iron depletion by DFO exhibited differential outcomes via a phase dependent manner.

The behind mechanisms responsible for these differential outcomes of iron depletion might depend on the different contributions of iron during the process of DSS-induced colitis. Anyhow, either inflammation or colonic barrier integrity would be finally reflected by the activity of colonic ISCs. Thus, we examined the two important signaling pathways in ISCs, including STAT3 and ERK. Accordingly, it was found that there was a significant loss of p-STAT3 and p-ERK1/2 positive signals within cryptal epithelial cells in the colon at day 5 after giving of DSS, and early DFO treatment reversed these decreases. However, late DFO treatment inhibited the recovery of these signals at day 10, especially p-ERK1/2 pathway (**Figure 6A**). We also evaluated the inflammatory immune cells on day 10. Interestingly, we observed mice in early DFO group had less infiltration of immune cells, such as MPO^+^ neutrophils, CD3^+^ T cells, and CX3CR1^+^/iNOS^+^ macrophages, indicating a greatly alleviated inflammation. The late DFO-treated mice had an infiltration similar to the mice received DSS only (**Figure 6B**). Immune cells, especially macrophages, are rich of iron, and iron level in these cells could promote them to secret pro-inflammatory chemokines and interleukins (52), which thus exaggerate colonic epithelial injury. In addition, iron can also regulate the polarization (53) and cellular function of macrophages (54). We infer that DFO-induced iron depletion might limit the hyperactivation of neutrophils and macrophages and decrease the content of ROS released by them, thus preserve the activity of p-STAT3 and p-ERK1/2 in ISCs. We are screening the secretion profile of DFO-treated macrophages in order to establish more specific molecule-cell-tissue interaction network during DSS-induced colitis. In the future research, more attention should be paid to the homeostasis of iron in colonic tissues. The appropriate combination with DFO and other current treatments in the early stage of acute colitis might be a new promising therapeutic method for the prevention and management of colitis. In the convalescent period, iron supplementation could increase intracellular iron concentration and promote the repair and regeneration of colonic mucosa. Of course, in this process, we need to make accurate judgments about the inflammatory stage of colitis, and at the same time strictly regulate the iron intake in the diet in order to treat it more accurately and effectively. So, we considered that iron depletion was a double-edge sword for the occurrence and recovery of experimental colitis, and the specific roles of iron metabolism during the whole process of colitis might explain the potential mechanisms.

## 5 Conclusion

In summary, this study demonstrated that iron depletion by DFO was beneficial to the onset of DSS-induced experimental colitis, but it limited the recovery of colonic epithelium from inflammation induced injury. Based on the current knowledge, this is the first investigation discussing the influence of iron ablation on ulcerative colitis. Our study emphasized that the manipulation of iron balance might be a potential therapeutic target for the prevention and treatment of ulcerative colitis in the clinical practice. We would further elucidate the specific roles of iron depletion on different subtype cells in the next study.

## Data Availability

The raw data supporting the conclusions of this article will be made available by the authors, without undue reservation.
