# Supplementary material for: Phase-dependent iron depletion differentially regulates the niche of intestinal stem cells in experimental colitis via ERK/STAT3 signaling pathway
Source: Front Immunol. 2025 Jan 30;16:1537651. doi: 10.3389/fimmu.2025.1537651 (PMC11822217; doi:10.3389/fimmu.2025.1537651)
Supplement: Supplementary file 1 [file DataSheet1.docx]

**Supplementary materials**

**Table S1. Primer sequences used for qPCR examination**

| **Gene** | **Forward primer** | **Reverse primer** |
| --- | --- | --- |
| Ms IL-1b | ACCTCACAAGCAGAGCACAA | TTGGCCGAGGACTAAGGAGT |
| Ms IL-10 | TAAGGCTGGCCACACTTGAG | GTTTTCAGGGATGAAGCGGC |
| Ms Lgr5 | CCTACTCGAAGACTTACCCAGT | GCATTGGGGTGAATGATAGCA |
| Ms Mki67 | ATCATTGACCGCTCCTTTAGGT | GCTCGCCTTGATGGTTCCT |
| Ms Ocln | TCCGGCCGCCAAGGTTC | CATAGCCTCTGTCCCAAGCAA |
| Ms Tjp1 | GCCTTGGCCTAGCATACACA | GGTAAGGCATTCCTGCTGGT |
| Ms TNF-a | ATGGCCTCCCTCTCATCAGT | TTTGCTACGACGTGGGCTAC |
| Ms Actb | GTGACGTTGACATCCGTAAAGA | GCCGGACTCATCGTACTCC |
| Hu Lgr5 | CTCCCAGGTCTGGTGTGTTG | GAGGTCTAGGTAGGAGGTGAAG |
| Hu Ocln | GACTTCAGGCAGCCTCGTTAC | GCCAGTTGTGTAGTCTGTCTCA |
| Hu Tjp1 | ACCAGTAAGTCGTCCTGATCC | TCGGCCAAATCTTCTCACTCC |
| Hu Actb | CATGTACGTTGCTATCCAGGC | CTCCTTAATGTCACGCACGAT |

**Figure legend**

**Figure S1. Different doses of DFO had similar protective effects at the early stage of DSS induced colitis.** (A) IF images of ZO-1 staining in different groups. (B) IF images of Occludin in colons of DSS control group and different DFO treated groups. (C) qPCR examination results for the mRNA expression levels of *Tjp1*, *OCLN* and *Lgr5* genes in Caco-2 cells. (D) Alcian blue staining images of colonic tissue in DSS control group and other different DFO groups. (E) PAS staining showed there were more PAS^+^ goblet cells in those DFO groups compared with DSS control group.

**
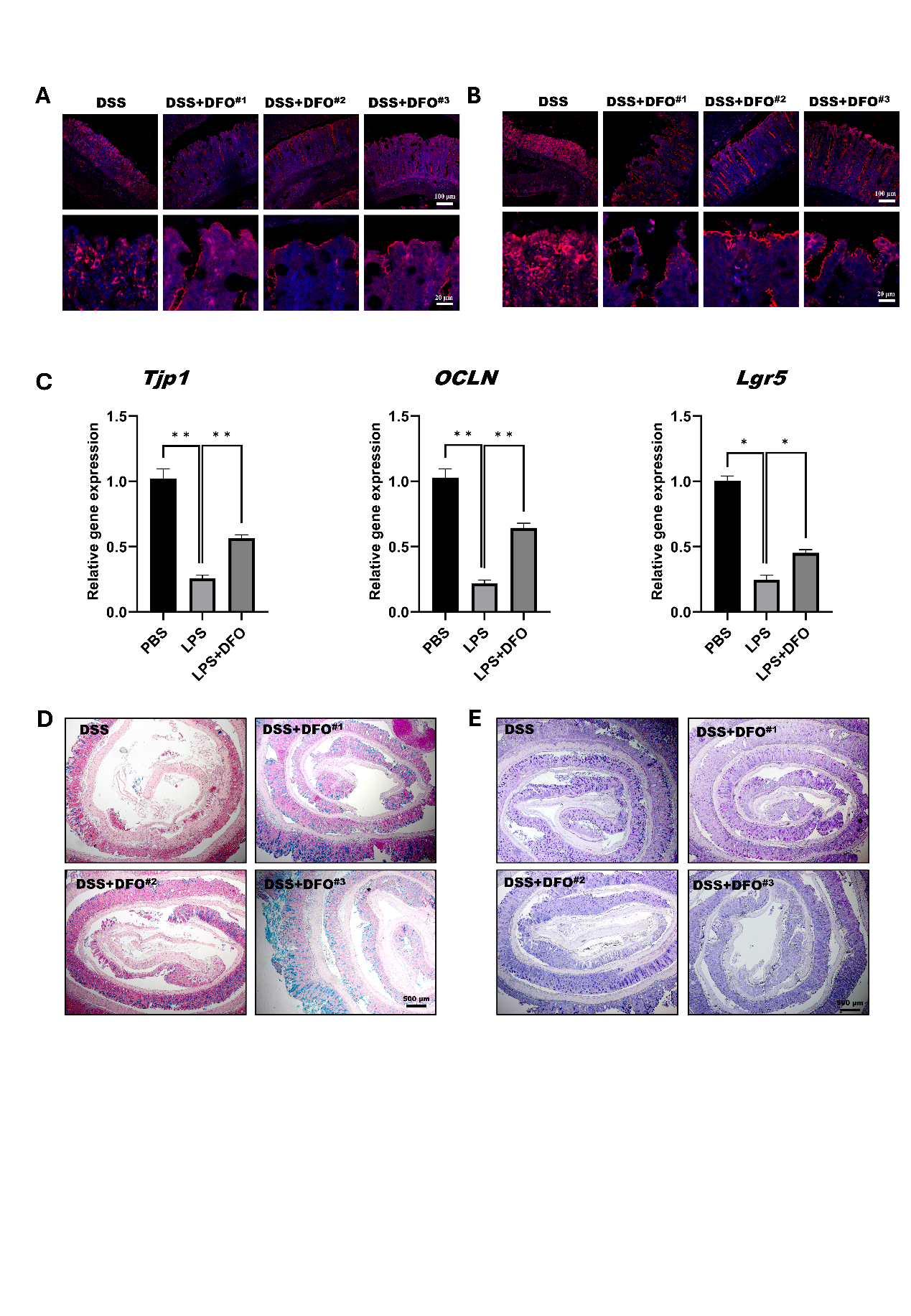
 Figure S1**
